# Correlation between Parameters of Calcaneal Quantitative Ultrasound and Hip Structural Analysis in Osteoporotic Fracture Patients

**DOI:** 10.1371/journal.pone.0145879

**Published:** 2015-12-28

**Authors:** Licheng Zhang, Houchen Lv, Hailiang Zheng, Ming Li, Pengbin Yin, Ye Peng, Yuan Gao, Lihai Zhang, Peifu Tang

**Affiliations:** 1 Department of Orthopedics, General Hospital of Chinese PLA, Beijing, China; 2 Department of Bioengineering, Beijing Shijitan Hospital, Capital Medical University, Beijing, China; University of Zaragoza, SPAIN

## Abstract

**Background:**

Calcaneal quantitative ultrasound (QUS), which is used in the evaluation of osteoporosis, is believed to be intimately associated with the characteristics of the proximal femur. However, the specific associations of calcaneal QUS with characteristics of the hip sub-regions remain unclear.

**Design:**

A cross-sectional assessment of 53 osteoporotic patients was performed for the skeletal status of the heel and hip.

**Methods:**

We prospectively enrolled 53 female osteoporotic patients with femoral fractures. Calcaneal QUS, dual energy X-ray absorptiometry (DXA), and hip structural analysis (HSA) were performed for each patient. Femoral heads were obtained during the surgery, and principal compressive trabeculae (PCT) were extracted by a three-dimensional printing technique-assisted method. Pearson’s correlation between QUS measurement with DXA, HSA-derived parameters and Young’s modulus were calculated in order to evaluate the specific association of QUS with the parameters for the hip sub-regions, including the femoral neck, trochanteric and Ward’s areas, and the femoral shaft, respectively.

**Results:**

Significant correlations were found between estimated BMD (Est.BMD) and BMD of different sub-regions of proximal femur. However, the correlation coefficient of trochanteric area (r = 0.356, *p* = 0.009) was higher than that of the neck area (r = 0.297, *p* = 0.031) and total proximal femur (r = 0.291, *p* = 0.034). Furthermore, the quantitative ultrasound index (QUI) was significantly correlated with the HSA-derived parameters of the trochanteric area (r value: 0.315–0.356, all *p*<0.05) as well as with the Young’s modulus of PCT from the femoral head (r = 0.589, *p*<0.001).

**Conclusion:**

The calcaneal bone had an intimate association with the trochanteric cancellous bone. To a certain extent, the parameters of the calcaneal QUS can reflect the characteristics of the trochanteric area of the proximal hip, although not specifically reflective of those of the femoral neck or shaft.

## Introduction

With the increase in the aging population, osteoporosis-related fractures are emerging as a major public health threat. Reports have shown that in patients older than 60 years of age, approximately 50% of women and 33% of men will suffer from osteoporosis-related fractures [1]. Osteoporosis-related fractures are associated with significant morbidity and mortality, especially in the case of hip fracture [2,3], with the 1-year mortality rate being approximately 27.6–40.5% for women and 15.8–23.3% for men [4]. Therefore, early evaluation of bone health status and the prompt diagnosis of osteoporosis are vitally important.

On account of its various advantages including low cost, simplicity of performance, and absence of radiation, calcaneal quantitative ultrasound (QUS), has recently been widely studied and found to have potential for osteoporosis diagnosis. Langton et al. first introduced the use of QUS for clinical bone mineral density evaluation in 1984 [5]. Since then, many clinical studies have found that certain QUS parameters, such as broadband ultrasound attenuation (BUA) and speed of sound (SOS), are significantly associated with fracture risk [6–8]. Furthermore, apart from the bone mineral density (BMD) and acoustic parameters, QUS examination can also provide mechanical information by means of calculating an index of stiffness, i.e., the quantitative ultrasound index (QUI). Huopio et al. found that the hazard ratio (HR) for fractures increased by 1.90 (95% CI, 1.25–2.91) per SD decrease in stiffness [9]. Based on a pooled meta-analysis of three prospective studies, Moayyeri et al. also determined that the BUA, SOS, and stiffness were significantly associated with fracture risk [10]. They reported that stiffness had the highest efficacy of fracture prediction, with HR of 2.26 (95% CI, 1.71–2.99) per SD decrease in stiffness. Specifically, several high-quality longitudinal studies have also demonstrated that calcaneal QUS can predict hip fracture risk in postmenopausal women [6,11,9]. Overall, recent studies have shown an intimate relationship between the parameters of calcaneal QUS and fracture risk.

However, osteoporosis-related fractures typically occur in the proximal femur, vertebrae, distal radius, and proximal humerus rather than in the calcaneus; therefore, whether is it suitable to use parameters from a non-osteoporotic fracture-affected site for the assessment of osteoporosis status at anatomically differing sites remains unclear. Bone structure and mechanical properties, which are predetermined by different local mechanical stimuli, vary from site to site [12,13]. Although both bones are of the cancellous type, cancellous bone from the calcaneus and proximal femur have their own distinctive structural and mechanical properties [14]. Indeed, a previous clinical study found an association between high calcaneal stiffness and low hip fracture risk [8]. However, few experimental studies have been performed to validate this association [15–19]. Furthermore, most of these experimental studies only focus on the association between calcaneal QUS and hip or spine dual energy X-ray absorptiometry (DXA) [16–19]. Bouxsein et al. used to report that heel QUS measurements were strongly correlated with strength of the proximal femur [20]. However, further associations between heel QUS measurements and hip sub-regions, namely femoral neck, trochanteric area, Ward’s area, femoral shaft, and total hip remain unclear. Since region-specific changes in the bone status may lead to different fracture risks and fracture types, understanding the association between calcaneal QUS and the different regions of the proximal femur could provide further information for the clinical evaluation of osteoporosis and fracture risk.

In this study, we aimed to explore the differences in the correlations of bone status between the calcaneus and the different regions of the proximal femur and to validate whether calcaneal stiffness was associated with the strength parameters, including the hip structural analysis (HSA) derived parameters and Young’s modulus of the primary load trabecular column in the femur. Quantifying the relationship between calcaneal stiffness as calculated by QUS, and the mechanical parameters obtained by the HSA and compression test are expected to help us further understand the value of QUS, by providing improved information for clinical assessment and decision-making.

## Materials and Methods

### Subjects

For this study, subjects were prospectively enrolled at the General Hospital of the Chinese People’s Liberation Army (PLAGH) in Beijing, China. Fifty-six elderly postmenopausal women (age, 56–95 years) with fragile femoral neck fractures, who were admitted at our institution from January 2014 to October 2014, were consecutively included in this study. However, three of the 56 femoral heads were destroyed during the surgery, and were, therefore, excluded from the analysis (S1 Dataset). All patients were diagnosed with fragility fracture and underwent either hemiarthroplasty or total arthroplasty. A fragility fracture was defined as spontaneous or as the consequence of a fall from standing height or lesser, according to the standard defined by the World Health Organization [21,22]. The study inclusion criteria were as follows: (1) women, age > 50 years old, (2) diagnosed with osteoporotic femoral neck fracture, (3) underwent hemiarthroplasty or total arthroplasty, and (4) postmenopausal patients. Patients with tumors, joint infections, diabetes, or any other diseases or who were using drugs that would influence bone metabolism, were excluded from the study. The baseline characteristics, such as age, years after menopause, weight, and height, of each patient were collected at the time of admission. Weight and height were measured without shoes or heavy outer clothing, using a standard stadiometer. The femoral diameter was measured precisely using medical engineering software (3-matic^®^ 6.0, Materialise NV, Leuven, Belgium). All patients were evaluated by QUS and DXA, and femoral head samples were collected and prepared for computer tomography (CT), quantitative computer tomography (QCT) scan and mechanical test. All patients signed an informed consent for study participation, and the study was approved by the medical ethics committee of the General Hospital of the Chinese People’s Liberation Army (PLAGH).

### Quantitative calcaneal ultrasound measurements (QUS)

QUS (Sahara Clinical Bone Sonometer, HOLOGIC, Bedford, MA, USA) was performed using a waterless device designed for the calcaneus of the fractured site, with the patient in the sitting position. The frequency of the ultrasound was 0.6 MHz and the peak-negative acoustic pressure was less than 1 MPa. Two parameters, the SOS and BUA, were automatically calculated by the system and were used for further calculation of the QUI and the estimated bone mineral density (Est.BMD). In particular, the QUI was calculated automatically using the equation “QUI = 0.41 × (BUA + SOS) − 571,” without a unit of measurement [23,24]. The Est.BMD (g/cm^2^) was calculated using the following equation “Est.BMD = 0.002592 × (BUA+ SOS)- 3.687” [25,26]. All ultrasound measurements were performed by the same investigator throughout the study. The Est.BMD was inferred from a linear combination of BUA and SOA, and not a direct measurement of the heel BMD. However, Est.BMD has proven to be a useful parameter for the assessment of calcaneal BMD in previous studies [25]. The coefficients of variation for the Est.BMD, SOS, BUA, and QUI were 3%, 0.22%, 3.7%, and 2.6%, respectively.

### Dual energy X-ray absorptiometry (DXA) and Hip structural analysis (HSA)

**Fig 1:**
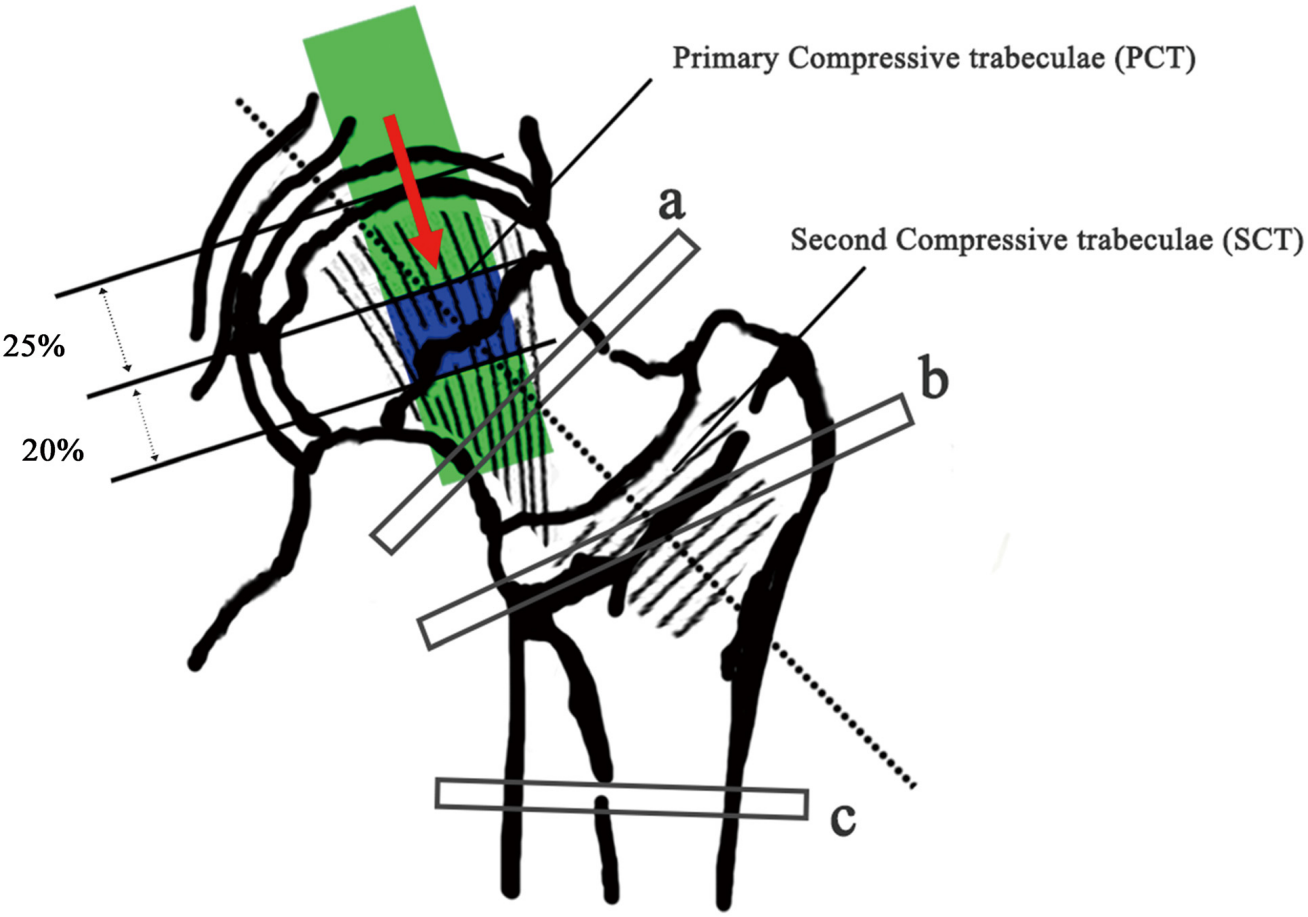
Weight-bearing system of the proximal femur and trabecular column extracted from QCT and mechanical test. The primary (PCT) and second compressive trabeculae (SCT) were delineated in the figure. After extracted from femoral head, PCT was cut into three segments. The first segment was ~25% of the diameter of the femoral head and the second segments was ~20%. The green box shows the extracting channel of the femoral head, while the blue box shows the 20% diameter length of the trabecular column that was used for the QCT scan and mechanical test. The red arrow indicated the loading direction of mechanical test. a, The femoral neck area of interest (NN); b, the trochanteric area of interest (IT); and c, the shaft area of interest (FS) during the analysis of BMD and HSA.

DXA (HOLOGIC Discovery-A, Apex software version 13.3) was performed at 3 days postoperatively. The investigated parameters included the BMD at the femoral neck, trochanteric region, and total hip, which were all generated automatically. The coefficient of variation for the total hip BMD was 0.8%. Using software provided by the DXA manufacturer, HSA was performed across the cross-section of three different sites (Fig 1), i.e., (1) the femoral neck (the narrowest point of the neck, NN), (2) trochanteric region (along the bisector of the neck shaft angle, IT), and the (3) femoral shaft (a site at a distance of 1.5 cm distal to the minimum neck width at the intersection of the neck and shaft axes, FS) (Fig 1). In this study, we used the following measurements at the above three sites: A. Cortical bone parameters, including the (1) sub-periosteal width (SubPeriWidth); (2) estimated endosteal width (EndoCortWidth); and (3) estimated cortical thickness (CortThick); B. Strength parameters, including the (4) cross-sectional area (CSA, index of resistance to axial forces); (5) cross-sectional moment of inertia (CSMI, estimate of resistance to bending forces in cross-section); (6) section modulus (*Z*, an index of the strength of the section); (7) Buckling ratio (BR, a variable indicated the loss of strength and higher BR indicates a precipitous loss of strength and may occur with local bucking) [27,28]. All the above parameters were evaluated at the three sites (NN, IT, and FS) and acronyms were expressed using the combination of sites with parameter acronyms, such as NN.SubPeriWidth, NN.EndoCortWidth, NN.CortThick etc. The first two letters (NN, IT or FS) indicate the site of evaluation while the other letters after the dot indicate the specific parameter that was measured.

### Specimen preparation

A self-designed sampling method combining X-ray tomography (CT) with three-dimensional printing (3D printing) was used for accurately locating and trephining the PCT columns. Details of this combined 3D-printing method have been described in our previous study [29]. Briefly, based on the CT scan data, the PCT column could be easily confirmed from three different planes (coronal, sagittal and horizontal planes) and a cylinder representing the sampling location was implanted in the primary stress trabecular arc. A bowl-shaped mold with needle channel was subsequently created with the femoral head concave and surface contour as reference. The designed mold was then exported in the stl format and printed in Transparent Fullcure^®^720 (OBJET EDEN 260V, Stratasys Ltd, Rehovot, Israel). Using this method, the primary trabecular columns could be correctly and easily extracted using a 12-mm diameter sterile trephine. After CT scan, samples were stored in dry conditions at -80°C before the subsequent tests. Each column was cut into three segments using a low speed saw (TechCut 4, Allied High Tech Inc., USA). The first segment was ~25% of the diameter of the femoral head (25% of the diameter length extending from the articular surface to beneath), and the second segments was ~20% (Fig 1). In our previous studies, the second segments were confirmed as the center of the femoral head, which is the densest part that bears the heaviest load [29]. Therefore, in this study, we only measured the mechanical properties of this second part.

### Quantitative computer tomography (QCT)

QCT scans of the extracted trabecular columns were obtained on a Brilliance iCT scanner (Brilliance iCT 728306, Philips, Netherlands). A hydroxyapatite reference phantom (QA calibration phantom, Mindways Software, Inc., USA) was placed beneath the sample for density measurement. Scans were performed at the following settings: 0.625 mm slice thickness, 512 × 512 pixels image matrix size, 480 mA X-ray tube current and 120 kVp voltage. Exposure time was about 600 ms. Radiation exposure was about 22 mGy/cm. The second segment of the extracted trabecular column was selected for calculation of the volumetric bone mineral density (BMD-QCT, mg/cm^3^) using software QCT Pro (QCT ProTM Mindways Software, Inc., USA).

### Mechanical tests of trabecular columns

After the QCT scans, vertical unconfined compression tests were performed for each column. Each column was compressed in the inferosuperior direction between two plates at the speed of 1%/min on Instron 3366 10 kN Dual Column Testing Systems (Instron, High Massachusetts, USA). The loading direction was consistent with longitudinal direction of the samples, as indicated in Fig 1. A stress-strain curve was plotted as the test progressed, and three parameters, including the Young’s modulus, as well as the yield and ultimate strengths, were calculated, in order to describe the mechanical properties of the trabecular columns.

### Statistical analysis

Data was expressed as mean ± standard deviation (SD). Normality of the distribution was assessed by the Kolmogorov-Smirnov test. Differences between the two groups were determined using the Student’s t-test. Pearson’s correlation analysis was performed to detect the potential association between QUS parameters (SOS, BUA, and QUI) and HSA-derived parameters, (CSA, CSMI, *Z* and BR) as well as the mechanical properties of the trabecular column (Young’s modulus, yield strength, and ultimate strength). All statistical analyses were performed using the SPSS 19.0 software (IBM Corporation, Armonk, NY), and a p value of < 0.05 was considered significant.

## Results

### Baseline characteristics

We included 53 female patients (age, 73.6±11.2 years) with osteoporosis-related femoral neck fractures in this study. The background data of subjects were shown in Table 1. The average number of years after menopause was 23±11 years, and 90.6% (45 of 53 patients) of the patients were older than 60 years old. The femoral neck BMD (T-score) was used to divide patients into two groups, as those with T≤-2.5 (N = 26) and T>-2.5 (N = 27), respectively. The average diameter of the femoral head was 5.0±0.4 cm. Bone data including calcaneal Est.BMD, BUA, QUI, and BMD of DXA differed significantly between the two groups.

**Table 1:** Background data of study subjects (n = 53; mean ± SD).

	T≤-2.5 (N = 26)	T>-2.5 (N = 27)	*p*
**Age (years)**	75.4±11.1	71.9±11.2	0.255
**Years after menopause (years)**	24.4±10.4	21.7±11.2	0.364
**Weight (cm)**	66.8±16.0	69.3±12.8	0.529
**Height (cm)**	165.7±8.8	169.5±9.6	0.146
**BMI (Kg/cm** ^**2**^ **)**	24.2±5.3	24.0±3.5	0.874
**Femoral head diameter (cm)**	4.90±0.40	5.11±0.39	0.057
**Est.BMD (g/cm** ^**2**^ **)**	0.278±0.071	0.332±0.074	0.009*
**SOS (m/s)**	1482.0±17.7	1491.7±20.2	0.019*
**BUA (dB/MH**_**2**_ **)**	47.5±11.0	55.7±11.0	0.009*
**QUI**	56.1±11.0	64.7±11.7	0.009*
**DXA-BMD(g/cm** ^**2**^ **)**			
Total hip (Total.BMD)	0.623±0.077	0.771±0.102	<0.001*
Femoral neck (Neck.BMD)	0.488±0.067	0.682±0.083	<0.001*
Intertrochanteric (Tra.BMD)	0.477±0.047	0.608±0.094	<0.001*
Internal (Inner.BMD)	0.752±0.134	0.897±0.125	<0.001*
Ward’s area (Ward.BMD)	0.349±0.155	0.535±0.142	<0.001*
**Young’s modulus (MPa)**	233.6±73.6	280.6±79.4	0.030*
**Yield strength (MPa)**	5.3±2.5	4.8±2.4	0.460
**Ultimate strength (MPa)**	6.6±3.1	6.3±2.9	0.711

Femoral neck BMD (T-score) was used to divide patients into two groups,

*p < 0.05.

### Correlation of the different bone densitometric parameters

**Fig 2:**
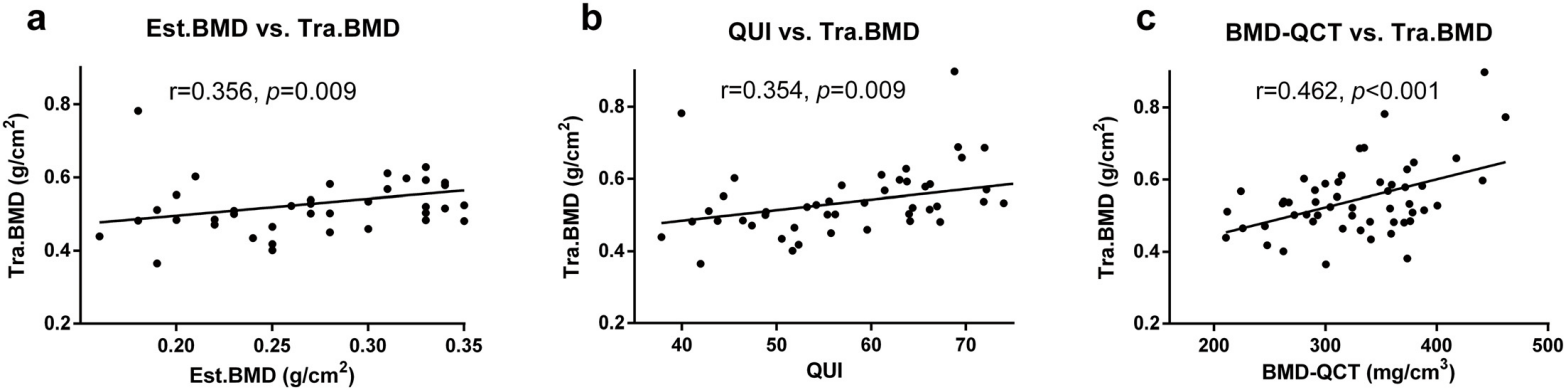
Pearson’s correlations between BMD values from the QUS, QCT, and DXA studies. The Est.BMD significantly correlated with the Tra.BMD (Fig 2a). The QUI significantly correlated with the Tra.BMD (Fig 2b). The BMD-QCT significantly correlated with the Tra.BMD (Fig 2c). The Tra.BMD indicates the BMD of the trochanteric area.

Pearson’s correlations between parameters from the QUS and BMD and from the QCT and DXA were shown in Table 2. Est.BMD determined by QUS was found to be significantly correlated with Neck.BMD (r = 0.297, *p* = 0.031), Tra.BMD (r = 0.356, *p* = 0.009) and Total.BMD (r = 0.291, *p* = 0.034). QUI was found to be significantly correlated with Neck.BMD (r = 0.297, *p* = 0.031), Tra.BMD (r = 0.354, *p* = 0.009) and Total.BMD (r = 0.287, *p* = 0.037) (Fig 2). SOS also significantly correlated with Neck.BMD, Tra.BMD and Total.BMD. While no significant correlations were found between BUA and BMD of different proximal femur sites.

**Table 2:** Pearson’s correlation of the acoustic parameters with the BMD of the proximal femur DXA

Variables	Neck.BMD	Tra.BMD	Inner.BMD	Total.BMD	Ward.BMD
**BUA (dB/MHz)**					
r	0.23	0.255	0.242	0.257	0.258
*p*	0.097	0.066	0.081	0.063	0.062
**SOS (m/s)**					
r	0.306*	0.377*	0.216	0.276*	0.195
*p*	0.026	0.005	0.121	0.045	0.162
**QUI**					
r	0.297*	0.354*	0.24	0.287*	0.233
*p*	0.031	0.009	0.083	0.037	0.093
**Est.BMD (g/cm** ^**2**^ **)**					
r	0.297*	0.356*	0.247	0.291*	0.231
*p*	0.031	0.009	0.075	0.034	0.096
**BMD-QCT (mg/cm** ^**3**^ **)**					
r	0.414*	0.462*	0.172	0.242	0.369*
*p*	0.002	<0.001	0.217	0.081	0.006

The unit of measurement for BMD from the DXA was g/cm^2^. Est.BMD was the estimated BMD value of the heel from the fracture side. BMD-QCT was the volumetric BMD value of the PCT column from the femoral head as obtained by the QCT scan. Neck.BMD, Tra.BMD, Inner.BMD, Total.BMD, and Ward.BMD indicate the BMD of the neck, trochanteric, inner, total and Ward’s area of the proximal femur, respectively.

*p < 0.05.

### Correlation between QUS-derived stiffness and HSA-derived parameters

**Fig 3:**
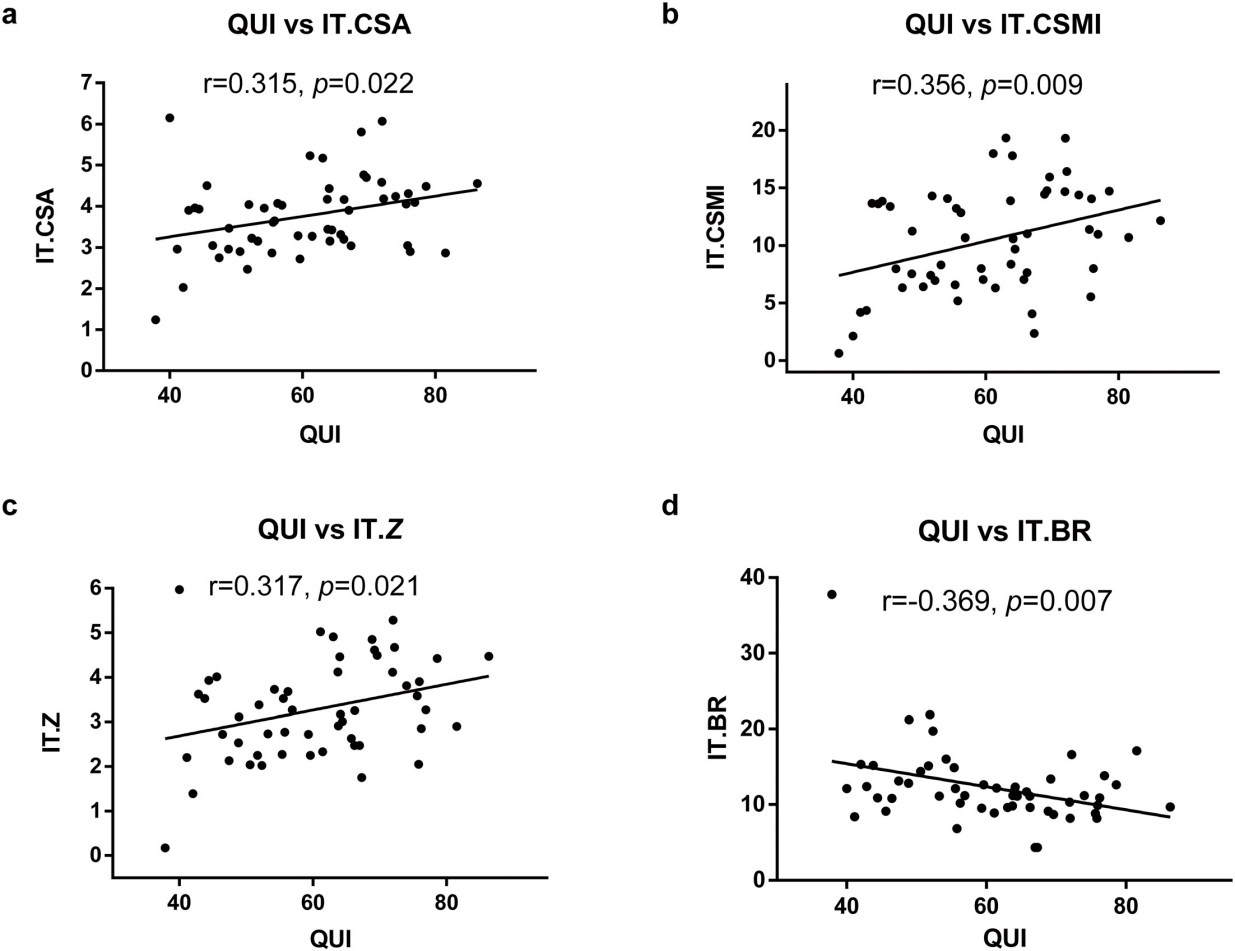
Pearson’s correlation of QUS-derived stiffness and HSA-derived parameters of the trochanteric area. The QUI was significantly correlated with IT.CSA (Fig 3a), IT.CSMI (Fig 3b), IT.*Z* (Fig 3c) and IT.BR (Fig 3d). The IT.CSA, IT.CSMI, IT.*Z* and IT.BR indicate the CSA, CSMI, *Z* and BR of the trochanteric area.

Table 3 shows the correlation of acoustic parameter QUI with the HSA findings for the proximal femur, as well as the correlation of the mechanical properties of the PCT trabecular column and the HSA of the proximal femur. QUI significantly correlated with parameters from the intertrochanteric area, namely IT.CSA (r = 0.315, *p* = 0.022), IT.CSMI (r = 0.356, *p* = 0.009), IT.*Z* (r = 0.317, *p* = 0.021) and IT.BR (r = -0.369, *p* = 0.007). Young’s modulus significantly correlated with IT.CSA (r = 0.338, *p* = 0.013), IT.CSMI (r = 0.447, *p* = 0.001), and IT.*Z* (r = 0.373, *p* = 0.006). In addition, QUI (r = 0.286, *p* = 0.038) and Young’s modulus (r = 0.322, *p* = 0.019) were also correlated with FS.*Z*. The yield and ultimate strengths did not show significant correlations with any of the HSA-derived parameters. The above relationship showed the trend that calcaneal acoustic parameter QUI were intimately associated with HSA-derived parameters in the trochanteric area, which contains a large amount of cancellous bone (Fig 3).

**Table 3:** Pearson’s correlation of the acoustic parameters and the mechanical test of the PCT with HSA analysis of the proximal femur.

	NN				IT				FS			
Item	CSA	CSMI	*Z*	BR	CSA	CSMI	*Z*	BR	CSA	CSMI	*Z*	BR
**QUI**												
r	0.164	0.084	0.167	-0.153	0.315*	0.356*	0.317*	-0.369*	0.22	0.122	0.286*	-0.168
*p*	0.241	0.551	0.233	0.275	0.022	0.009	0.021	0.007	0.114	0.384	0.038	0.23
**Young’s modulus (MPa)**												
r	0.184	0.151	0.197	-0.234	0.338*	0.447*	0.373*	-0.219	0.211	0.217	0.322*	0.003
*p*	0.188	0.279	0.157	0.092	0.013	0.001	0.006	0.116	0.13	0.119	0.019	0.983
**Yield strength (MPa)**												
r	-0.131	-0.054	0.014	-0.134	-0.027	0.031	0.005	0.039	-0.085	0.073	0.032	0.106
*p*	0.351	0.701	0.922	0.338	0.85	0.826	0.973	0.783	0.543	0.603	0.819	0.449
**Ultimate strength (MPa)**												
r	-0.109	-0.033	0.036	-0.126	-0.003	0.042	0.024	0.014	-0.069	0.081	0.049	0.089
*p*	0.437	0.816	0.8	0.371	0.985	0.768	0.865	0.921	0.624	0.566	0.728	0.525

The NN.CSA, NN.CSMI, NN.*Z* and NN.BR indicate the CSA, CSMI, *Z* and BR of the femoral neck area, respectively. The NN, IT, and FS indicate the femoral neck, trochanteric area, and femoral shaft, respectively. CSA: cross-sectional area; CSMI: cross-sectional moment of inertia; *Z*: section modulus; BR, Buckling ratio.

* *p* < 0.05.

Apart from the strength parameters, the HSA also provided certain parameters for cortical bone evaluation (Table 4). In this study, QUI (r = 0.371, *p* = 0.006), SOS (r = 0.387, *p* = 0.004) and BUA (r = 0.277, *p* = 0.044) were significantly correlated with the IT.CortThick, while QUI (r = 0.295, *p* = 0.032) and SOS (r = 0.341, *p* = 0.013) were significantly correlated with the NN.CortThick. These results indicated that the calcaneal acoustic parameters, which were not tailored for cortical bone evaluation, did not show strong numeric associations with the HSA-derived cortical parameters, except for NN.CortThick and IT.CortThick.

**Table 4:** Pearson’s correlation of the acoustic parameters with the HSA of the proximal femur cortical measurement

Item	NN.SubPeriWidth	NN.EndoCortWidth	NN.CortThick	IT.SubPeriWidth	IT.EndoCortWidth	IT.CortThick	FS.SubPeriWidth	FS.EndoCortWidth	FS.CortThick
**BUA (dB/MHz)**									
r	-0.062	-0.077	0.168	-0.114	-0.16	0.277*	0.011	-0.056	0.189
*p*	0.659	0.582	0.23	0.415	0.252	0.044	0.939	0.693	0.175
**SOS (m/s)**									
r	-0.114	-0.149	0.341*	0.08	0.02	0.387*	-0.024	-0.096	0.247
*p*	0.417	0.286	0.013	0.57	0.89	0.004	0.862	0.494	0.075
**QUI**									
r	-0.101	-0.131	0.295*	0.009	-0.05	0.371*	-0.013	-0.087	0.241
*p*	0.472	0.351	0.032	0.95	0.722	0.006	0.928	0.535	0.082

The NN, IT, and FS indicate the femoral neck, trochanteric area, and femoral shaft, respectively. SubPeriWidth, sub-periosteal width; EndoCortWidth, estimated endosteal width; CortThick, estimated cortical thickness.

* *p* < 0.05.

### Correlation between QUS-derived stiffness and mechanical properties of the principal compressive trabecular (PCT) column in the femoral head

**Fig 4:**
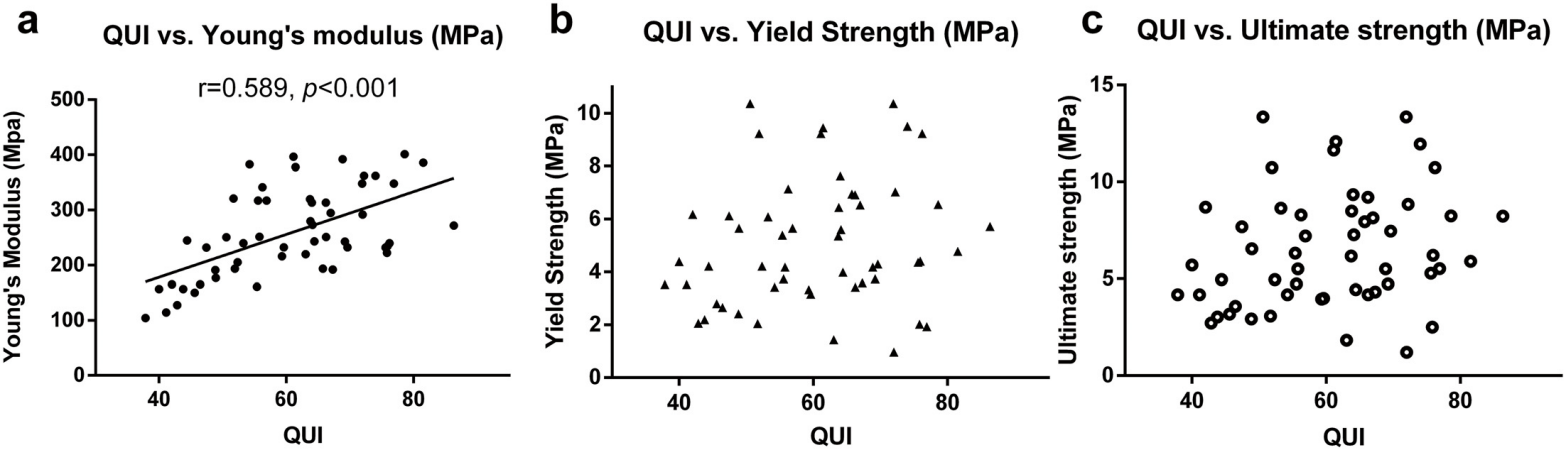
Pearson’s correlation of stiffness with mechanical properties of the primary compressive trabecular (PCT) column in the femoral head. The QUI significantly correlated with Young’s modulus of the PCT column of the femoral head (Fig 4a). However, no significant correlation was noted with the yield strength (Fig 4b), or ultimate strength (Fig 4c).

The PCT column of the femoral head, which conducts the mechanical load of weight to the femoral neck and femur, plays an important role in the load conduction process. Due to the inaccessibility of human proximal femur samples in this study, we used the PCT column of the femoral head as an alternative. The QUI was significantly correlated with the Young’s modulus of the PCT column of the femoral head (r = 0.589, *p*<0.001) (Table 3). However, no significant correlation with yield strength (r = 0.182, *p* = 0.192) or ultimate strength (r = 0.230, *p* = 0.098) was noted (Fig 4).

## Discussion

The primary result of our study was that Est.BMD and stiffness from the QUS significantly correlated with the HSA-derived parameters of the trochanteric area and with the Young’s modulus of PCT from the femoral head. The above results indicated that as both bones were of the cancellous type, the parameters of calcaneal QUS could reflect the bone mass and mechanical properties of the trochanteric area, to a certain extent.

According to its structural and functional differences, the proximal femur can be divided into different areas, such as femoral head, femoral neck, trochanteric area, Ward’s area, and femoral shaft. With changes caused by aging or disease, differences may occur among these areas in terms of the dynamic changes of bone rarefaction. Some studies demonstrated that the BMD value of the femoral head was higher than that of the trochanteric and Ward’s areas [30], while other studies have found that the bone loss rates differ among the different sites [31,32]. Thus, it is reasonable to presume that different changes between the abovementioned regions of the proximal femur may contribute differently to the mechanical properties and even the fracture risk. In clinical practice, the DXA and QCT can help detect regional differences in the abovementioned areas to provide detailed information. However, calcaneal QUS can only be used to evaluate the bone status of one place, i.e., the heel. Since calcaneal QUS is an easy and advantageous investigative technique, it is important to identify the part of the proximal femur that shows the closest correlation with calcaneal QUS in order to improve the clinical application and osteoporosis status evaluation offered by calcaneal QUS.

In this study, we found that Est.BMD from QUS significantly correlated with BMD of different sub regions of proximal femur. However, the Pearson’s correlation coefficient of trochanteric area (r = 0.356) was higher than that of the neck area (r = 0.297) and total proximal femur (r = 0.291). In addition, and QUI from QUS was also significantly correlated with the estimated mechanical parameters derived from HSA of the trochanteric area, such as IT.CSA (r = 0.315), IT.CSMI (r = 0.356), IT.*Z* (r = 0.317) and IT.BR (r = -0.369). Trimpou et al. evaluated the association of QUS with hip BMD in different areas in osteoporosis patients (BMD lower than -2.5 T-score by DXA) [19], and found that stiffness had the highest Pearson correlation value with BMD from the trochanteric area (r≈0.46, *P*<0.001) compared to BMD from the lumbar spine, distal radius, proximal radius, and femoral neck (r value ranging from 0.23 to 0.45). Our results were consistent with those of Trimpou et al. in terms of BMD evaluation (Table 2). On the other hand, our study also found a significant correlation between the calcaneal QUI and HSA-derived parameters in the trochanteric area, which has not been reported previously. These results indicated that calcaneal QUI could reflect the mechanical strength of the trochanteric area to some extent.

However, as HSA-derived parameters are all calculated based on the measurement of proximal cortex [33], the abovementioned correlation of calcaneal QUI with CSA, CSMI, and section modulus might not reflect the true relationship between QUI and the mechanical properties of trochanteric cancellous bone. A direct evaluation of the mechanical properties of trochanteric cancellous bone is necessary before drawing such conclusions. However, because of the inaccessibility of human proximal femur samples, alternative candidates need to be used for further evaluation. The trochanteric area represents the second compressive trabeculae; therefore, we chose the cancellous bone that was most similar, i.e., the PCT column of the femoral head, as the candidate for evaluation of Young’s modulus for trochanteric cancellous bone. The feasibility of using the PCT as a substitute, was supported by the similar BMD and high correlation of BMD between the extracted PCT column and trochanteric area (r = 0.462, *p*<0.001). In addition, in order to extract the exact PCT trabeculae, a new method combining CT and 3D-printing techniques was used, which has been described in detail previously [29]. Our results showed a significant correlation between QUI and Young’s modulus of PCT (r = 0.589, *p*<0.001) (Fig 4). Previously, Lochmüller et al. analyzed the correlation of calcaneal QUS parameters with mechanical failure loads of the femur and lumbar vertebral bodies; they found a significant association between QUI and mechanical failure loads of the femur (r = 0.49 for women and r = 0.55 for men). Our results were consistent with those reported by Lochmüller et al., with some numerical differences. We consider that the differences in the study population and measurement techniques have contributed to the partial differences between these results. Overall, calcaneal QUI potentially and partly reflects the mechanical properties of the trochanteric area. This can be illustrated by the results of the HSA-derived parameters and mechanical tests, as shown in this study.

Our study has certain limitations. First, all our results were calculated from samples of patients with femoral neck fracture. It is known that the structural and mechanical properties of bone may differ between patients with femoral neck fractures and intertrochanteric fractures [34]. The conclusion of this study should be confined to the fragile femoral neck fracture population. If our result is extrapolated to a patient population with intertrochanteric fractures, the association between the two methods, i.e., calcaneal QUS and DXA/HSA might be underestimated. The results of this study need to be evaluated in other population, such as normal, osteopenia and moderate osteoporosis population. Second, due to the inaccessibility of trochanteric bone samples, we used the PCT columns from the femoral head as an alternative. Third, the platens compression test used in this study has systematic and random errors due to end-artifacts [35], so future studies using more accurate mechanical testing methods are needed to confirm the conclusion of this study. Fourth, the small sample size is another limitation; in order to clarify and validate our findings, studies with a large sample size and with different populations are necessary. In particular, the correlation between QUI and HSA-derived mechanical properties could be retrospectively reanalyzed using data from previous large cohort studies, such as the Japanese Population-based Osteoporosis (JPOS) Study [26,36] and the ESOPO study [37].

## Conclusion

Our results showed that, as a cancellous bone that experiences weight loading, the calcaneal bone shows an intimate association with trochanteric cancellous bone. Thus, parameters of calcaneal QUS can, to a certain extent, reflect the characteristics of the trochanteric area of the proximal hip, although not specifically reflective of those of the femoral neck or shaft.

## Supporting Information

S1 Dataset Data of 53 patients used in this study. (SAV)
